# Surgical Removal of an Orthodontic Mini-Screw Displaced Into the Lateral Pharyngeal Space: A Case Report and Review of Pertinent Literature

**DOI:** 10.7759/cureus.52343

**Published:** 2024-01-15

**Authors:** Sameh A Seif, Yaser AlNatheer, Lama Al Bahis, Sundar Ramalingam

**Affiliations:** 1 Department of Oral and Maxillofacial Surgery, College of Dentistry, King Saud University, Riyadh, SAU; 2 Department of Oral and Maxillofacial Surgery, Faculty of Dentistry, Cairo University, Cairo, EGY; 3 Department of Oral and Maxillofacial Surgery and Diagnostic Sciences, College of Dentistry, Majmaah University, Majmaah, SAU

**Keywords:** fluoroscopy, parapharyngeal space, lateral pharyngeal space, foreign body displacement, temporary anchorage device, orthodontic mini-screw

## Abstract

Oral and maxillofacial surgeons are often faced with the clinical challenge of foreign body displacement into the perioral tissues and soft tissues of the head and neck. This mainly occurs either because of trauma or inadvertently during dental treatment. In addition to the maxillary sinus, iatrogenic foreign body displacement during dental treatment could happen into one of the 16 distinct fascial spaces of the head and neck region. Commonly displaced foreign bodies related to dental treatment include tooth roots or fragments, local anesthetic needles, implants and restorations. The clinical sequelae of a displaced foreign body depend on its size, shape, anatomic location and proximity to vital structures. Although patients may remain asymptomatic for a considerable amount of time, retained foreign bodies result in persistent pain, recurrent infection and scarring of soft tissue due to inflammation, all of which may complicate delayed retrieval. In addition to the history, imaging modalities such as plain radiographs and computed tomography (CT) help in locating the displaced foreign body and its subsequent retrieval. Surgical retrieval may be attempted through intraoral, transcervical and endoscopic approaches. Additionally, surgery may be aided by real-time imaging such as fluoroscopy. The present report aims to detail a case of inadvertent displacement of an orthodontic mini-screw, commonly used as a temporary anchorage device (TAD), into the lateral pharyngeal space, while attempting placement in the mandibular retromolar area. The case report also describes the surgical retrieval procedure of the TAD screw using an intraoral approach and with fluoroscopy guidance using C-Arm radiographic imaging. This case is reported along with the pertinent review of literature, as it not only explains a rare complication of orthodontic mini-screw placement but also details a modality to remove displaced foreign bodies from fascial spaces of the head and neck, which are otherwise directly inaccessible.

## Introduction

Orthodontic mini-screws are temporary anchorage devices (TAD) used for obtaining skeletal anchorage in cases where large forces need to be applied. A few common indications for the use of these mini-screws include orthodontic distalization, mesialization, intrusion and arch correction [1]. These mini-screws are capable of self-drilling and self-tapping, and unlike dental implants, they do not require osseointegration prior to mechanical loading. Conventionally, these mini-screws are applied onto the bone surface through mucosal puncture and are screwed in place by the application of pressure [1,2]. The retromolar area in the posterior mandible is an anatomical site where TAD mini-screws are frequently placed to distalize the mandibular molars [1]. In spite of their relative ease of use, utmost care needs to be exercised during clinical placement of orthodontic mini-screws in order to avoid complications [2,3].

It is not uncommon for maxillofacial surgeons to deal with foreign bodies, which are displaced into the oral cavity soft tissues, maxillary sinuses, the neck spaces, or any other adjoining anatomical structures of the head and neck area [4]. The head and neck region is anatomically complex and comprises several intricate and sensitive anatomical structures, including cranial nerves, major blood vessels, the aero-digestive tracts, and the auditory and visual apparatuses [5]. Injuries to these areas frequently involve the risk of foreign body entrapment, owing to their close proximity and greater exposure to the external environment [6]. Foreign body penetration and displacement into the tissues of the craniomaxillofacial region either could be unintentional, self-inflicted, iatrogenic, caused by assault, inhaled, swallowed or occur because of direct trauma [5,7].

Anatomically, there are 16 distinct fascial spaces located in the head and neck area. These are classified into four main subtypes: (1) Fascial spaces of the face - canine, buccal, parotid, infratemporal, and masticatory (masseteric, pterygomandibular, temporal); (2) Suprahyoid fascial spaces - sublingual, submental, submandibular, lateral pharyngeal/parapharyngeal, and peritonsillar; (3) Infrahyoid fascial spaces - pretracheal and paratracheal; (4) Fascial spaces of the neck - retropharyngeal, prevertebral (danger space), and carotid sheath [8]. Oral surgical and dental procedures in the posterior mandibular area such as local anesthetic injection, extraction of lower third molars, insertion of screws in the retromolar area, or dental implant placement can predispose to the inadvertent displacement of foreign bodies (teeth or implants) into three main fascial spaces: pterygomandibular space, submandibular space, or lateral pharyngeal space [4]. The pterygomandibular space is bounded anteriorly by the anterior border of the mandibular ramus and the pterygomandibular raphe. Posteriorly, it is bounded by the parotid gland capsule as it traverses from superficial to deep around the posterior border of the mandibular ramus. While the medial and lateral boundaries of the pterygomandibular space are formed by the medial surface of ramus and medial pterygoid muscle, respectively, the lateral pterygoid muscle lies superiorly and pterygomasseteric sling lies inferiorly [8]. The pterygomandibular space contains several vital anatomic structures including inferior alveolar nerve and blood vessels, along with the lingual nerve and nerve to mylohyoid and anterior belly of digastric [8].

The submandibular space is a triangular space bounded anteriorly by the anterior belly of digastric, posteriorly by the posterior belly of the digastric along with stylohyoid muscle and the digastric tendon forming the apex. Its superior boundary is formed by the inferomedial surface of mandibular body and the mylohyoid muscle, and the inferior boundary is formed by the investing layer of deep cervical fascia covering the hygolossus and mylohyoid muscles. Laterally and superficially, the submandibular space and its contents including facial artery and vein, marginal mandibular nerve, nerve to mylohyoid, and the submandibular gland and lymph nodes are covered by superficial cervical fascia and platysma muscle [8]. Unlike the aforementioned two spaces, which lie in close proximity to the mandibular bone, the lateral pharyngeal space, also known as the parapharyngeal space or pharyngomaxillary space, is situated in the upper neck region and lies posterior to the mandible. It is bounded anteriorly by pterygomandibular raphe, posteriorly by prevertebral division of deep cervical fascia along with the carotid sheath, posterolaterally, superiorly by the skull base (sphenoid bone) and inferiorly by the hyoid bone. Its medial and lateral boundaries are respectively made up of the visceral division of the deep cervical fascia (buccopharyngeal fascia) encasing the pharyngeal constrictors and the superficial layer of the deep cervical fascia overlying the mandible, medial pterygoid, and parotid gland. Important anatomic structures housed by the lateral pharyngeal space include the ninth (glossopharyngeal), 11th (accessory) and 12th (hypoglossal) nerves, carotid sheath, part of the cervical sympathetic chain, and lymph nodes [8].

Most cases of foreign body displacement associated with dental or oral surgical treatment in the posterior mandible are reported in the pterygomandibular and submandibular spaces, owing to their anatomic proximity to the mandible [4]. Foreign body displacement to the lateral pharyngeal space occurs rarely and is predominantly due to deeper migration of a dislodged foreign body, left in situ in one of the superficial anatomic spaces, and occurs because of jaw movements [9]. Radiographic imaging modalities play a significant role in anatomic localization of foreign bodies and also during imaging-assisted surgical procedures for their retrieval [7]. While traditional methods involved the use of two or more radiographs taken in different planes to three-dimensionally (3D) locate the dislodged foreign body, computed tomography (CT) and ultrasonography (USG) are used contemporarily, and are superior to plain radiographs, for anatomic localization [6]. In this regard, fluoroscopy (C-Arm mobile imaging system), a real-time imaging modality that uses X-rays for visualizing movements of instruments and implants within body parts during surgery, has found an application in surgical retrieval of foreign bodies. In addition to helping visualize the dislodged foreign body in real-time, C-Arm fluoroscopy has also enabled indirect yet guided access to the fascial spaces in the head and neck, which are anatomically complex due to their location and associated structures [9-11].

This paper aimed to report on a case of inadvertent displacement of an orthodontic mini-screw into the lateral pharyngeal space, while trying to place it in the posterior mandible, and subsequent surgical retrieval of the foreign body, guided by intraoperative C-Arm fluoroscopy. In addition, this report details an unusual complication, which could be encountered during placement of implants and TADs in the posterior mandible and how to manage it surgically.

## Case presentation

**Figure 1:**
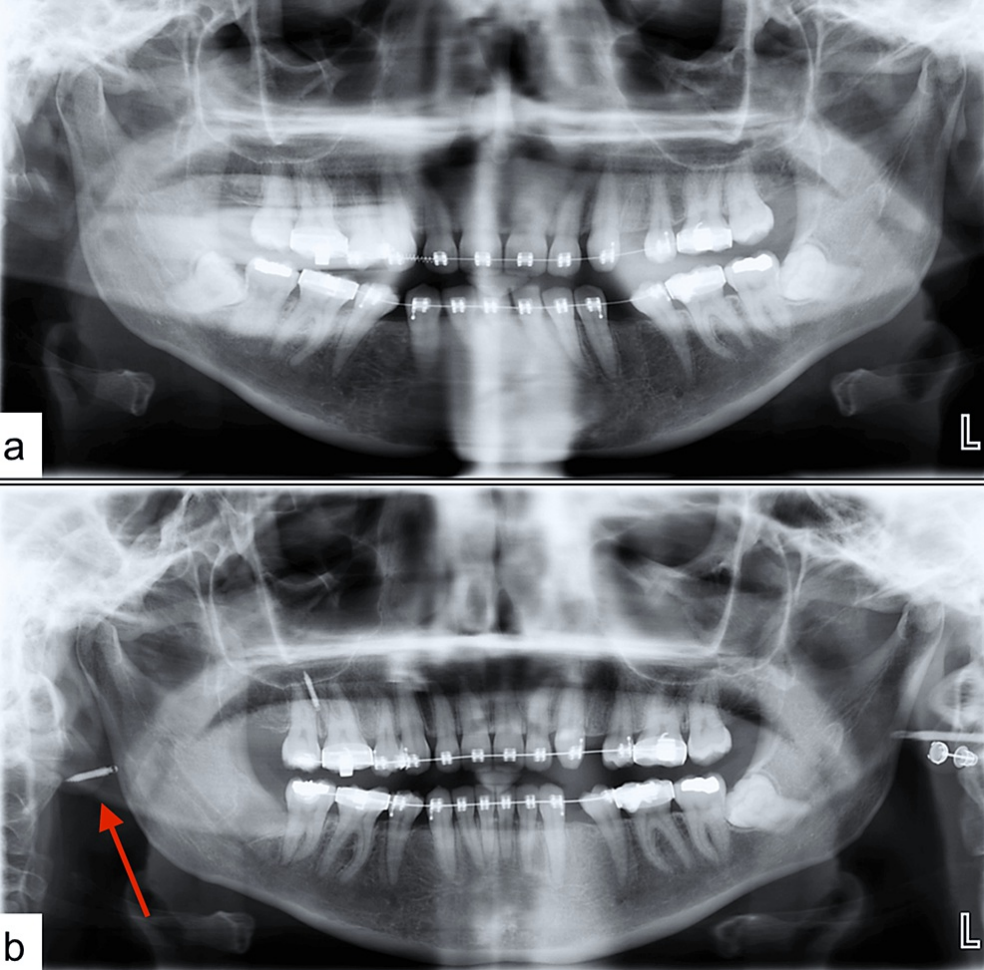
(a) Preoperative orthopantomogram (OPG) showing extracted maxillary third molars (18 and 28) and impacted mandibular third molars (38 and 48); (b) OPG taken after dislodgement of the mini-screw showing extraction socket of tooth 48 and mini-screw displaced into the soft tissues posterior to the ramus of the right mandible

**Figure 2:**
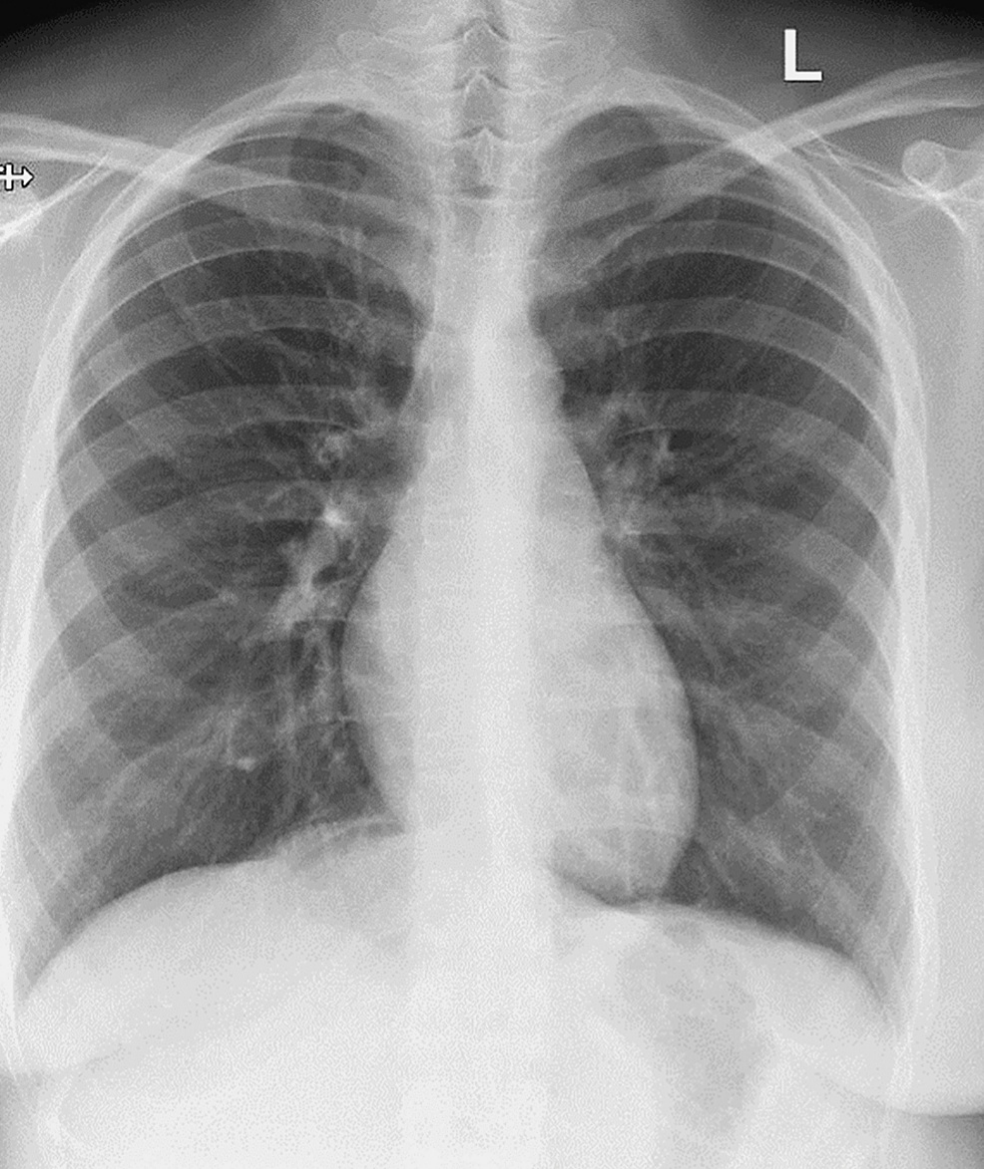
Unremarkable chest radiograph

**Figure 3:**
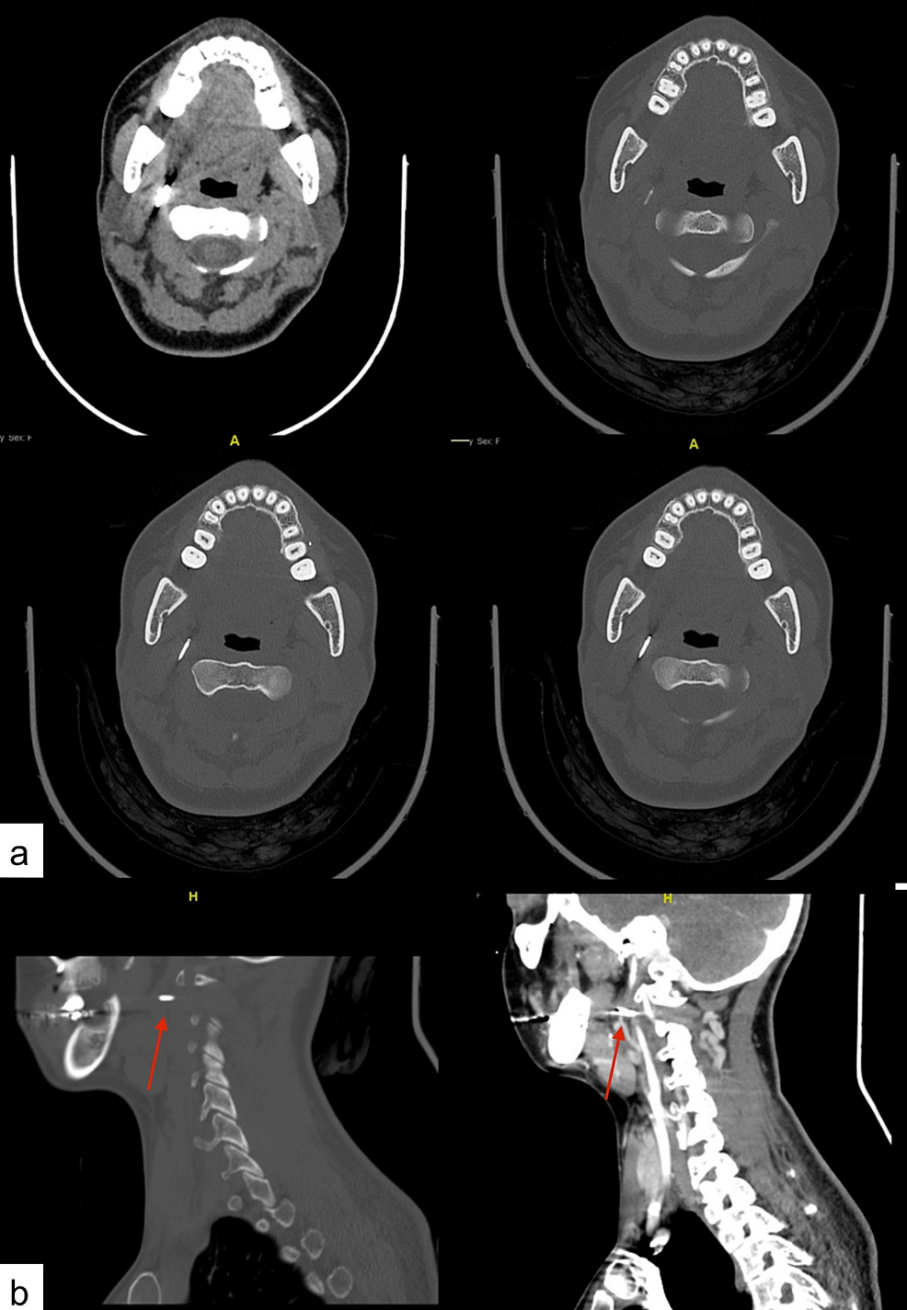
(a) Axial sections of head and neck computed tomography (CT) showing the mini-screw located within the right lateral pharyngeal space and in close proximity to the major blood vessels of the neck; (b) Sagittal sections of head and neck CT showing the screw in the right lateral pharyngeal space

A 30-year-old female patient, with no reported medical illnesses or allergic conditions, was referred to the oral and maxillofacial surgery clinic for extraction of the right mandibular third molar (tooth #48) and subsequent placement of orthodontic mini-screws (TAD) (Figure 1A). The orthodontist had requested placement of TAD at two sites, one in the right maxillary molar region and another in the right posterior retromandibular area. The TADs were respectively indicated for skeletal anchorage and distalization of the right mandibular second molar. The patient had a previous history of dental extractions for the upper third molars and all first premolars, under local anesthesia and was comfortable during the procedure, except for mild apprehension, due to the first time to undergo TAD placement. Maxillary TAD placement and extraction of the right mandibular third molar progressed uneventfully. However, during placement of the mandibular TAD the patient made a sudden mandibular movement due to sharp pain, resulting in the mini-screw being displaced from the surgical site and dislodged into soft tissue medial to the ramus of the mandible. Initial attempts to identify the displaced mini-screw through examination and palpation ended in failure. After reassuring the patient and making sure that she did not present with breathing difficulties, dysphagia, regurgitation, or hiccups, orthopantomogram (OPG) and chest radiograph were ordered. While the chest radiograph was unremarkable (Figure 2), OPG revealed the dislodged mini-screw in the soft tissues posterior to the ramus of the right mandible (Figure 1B). This was followed by a contrast-enhanced CT scan of the head and neck region for the 3D location of the dislodged mini-screw. CT images showed the foreign body dislodged within the right lateral pharyngeal space and close to major blood vessels of the neck (Figure 3). The patient was subsequently informed about admission and surgical retrieval of the mini-screw under general anesthesia (GA) and agreed to the same.

**Figure 4:**
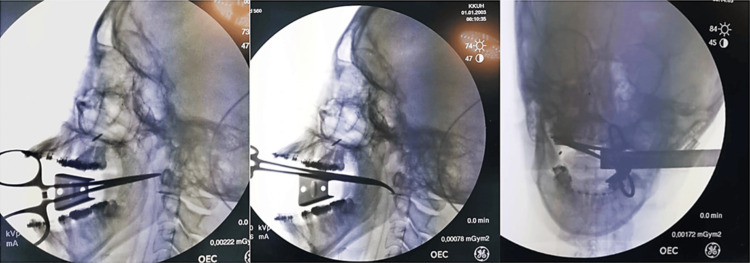
Intraoperative fluoroscopy using the C-Arm radiographic imaging system in multiple planes (from left to right – lateral, oblique and anteroposterior) showing the location of the mini-screw in the right lateral pharyngeal space and with respect to the tip of the hemostat

**Figure 5:**
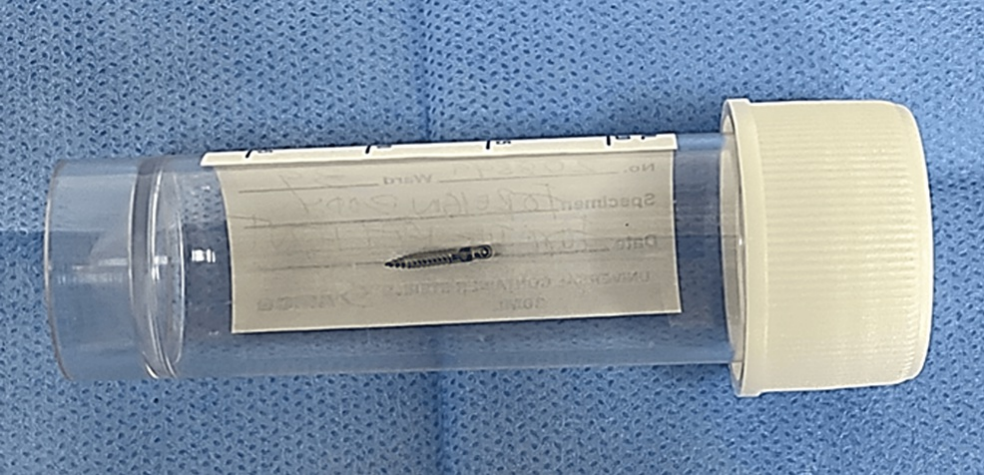
The mini-screw after surgical retrieval

Informed consent was obtained from the patient, after explanation of the outcomes, risks and complications, for surgical site exploration and removal of the foreign body under general anesthesia. Following GA induction and nasal endotracheal intubation, the head and neck area was prepared and draped using aseptic protocol. Intraoral irrigation with chlorhexidine was done and local anesthesia with 2% lidocaine and epinephrine (1:100,000) was administered in the right pterygomandibular space, buccal vestibule and retromandibular area. Using the mucosal entry point of the TAD mini-screw as a guide, an incision was made using electrocautery, with the surgical approach similar to that of bilateral sagittal split ramus osteotomy. After mucoperiosteal flap elevation, blunt dissection was carried out medial to the ramus and towards the angle of the mandible, using dissecting forceps and finger dissection. With the dissecting forceps in situ, multiple fluoroscopy images in the lateral, oblique and anteroposterior planes were obtained using a portable C-Arm X-ray machine to locate the position of the screw (Figure 4). After guidance and confirmation through imaging, the foreign body was retrieved successfully (Figure 5). Hemostasis in the surgical site was achieved through packing and application of absorbable powder hemostatic agent (AristaTM AH Absorbable Hemostatic Particles; BD, Franklin Lakes, NJ, USA) and closure was done using Vicryl 3-0 absorbable sutures (Polyglactin 910; ETHICON, Somerville, NJ, USA). Following an uneventful immediate postoperative period, the patient was discharged the day after surgery and was advised to follow up in the maxillofacial surgery clinic after two weeks. During the recall visit, the patient reported no active complaints or neurosensory deficiency and was discharged from the maxillofacial clinic in a stable condition, along with a referral for continuing orthodontic treatment.

## Discussion

Dislodgement of foreign bodies into the soft tissues of the head and neck region is a complication, which may occur in patients of all age groups and could be due to various causes [4]. In most cases, they are either traumatic in origin, such as after road traffic accidents, gunshot wounds, shrapnel from explosives, and industrial accidents, or they could be iatrogenic, such as broken medical instruments, dental local anesthetic needles and dislodged implants [7]. Dental treatment is associated with the risk of foreign body displacement into perioral soft tissues and rarely into deeper head and neck spaces. While these are mostly preventable by diligent adherence to protocol, they still occur frequently due to a challenging intraoral work environment and inadvertent slippage of instruments due to difficult access [4]. Displaced head and neck foreign bodies could vary in size, shape, anatomic location and proximity to vital structures. Although patients may remain asymptomatic in the immediate aftermath of foreign body dislodgement into head and neck tissues, they are potentially infectious and can put the patient’s life at risk. Especially, iatrogenic foreign body dislodgement of dental instruments, needles and implants carry with them the risk of contamination from oral microbial flora [4,12]. It is therefore advisable to remove any head and neck foreign body to avoid recurrent infections, foreign body reactions and the risk of damage to vital vascular and neurological structures [12].

It has been reported that foreign bodies that are left untreated end up causing local inflammatory reactions, persistent pain and scarring, thereby making delayed retrieval difficult [4,7]. Additionally, foreign bodies dislodged in the deep neck spaces could lead to hearing loss, dysphagia and trismus. Some of the absolute indications for head and neck foreign body removal include risk of airway obstruction, potential for damaging neurovascular structures and the vulnerability to cause scarring around muscles of mastication and facial expression. On the contrary, extensive surgical exploration leading to wound contamination, endangering orofacial function and esthetics, and risk of damaging vital anatomic structures comprise the rare contraindications for foreign body removal [7]. In the present case, the decision to remove the dislodged mini-screw without further delay was attributed to the fact that the screw was contaminated by saliva and because of its close proximity to major blood vessels of the neck, as shown by CT imaging.

With respect to managing head and neck foreign body displacement, it is imperative to arrive at a proper diagnosis, which would form the basis for the treatment plan. A comprehensive medical history combined with thorough clinical examination is the primary requirement to arrive at a diagnosis. In addition, the medical and social history of the patient should not be discounted, as most surgical retrievals require general anesthesia, owing to the need for ample surgical access and intricate surgical site manipulation [5,10]. In scenarios wherein the foreign body displacement is witnessed clinically, imaging modalities play the role of the next important diagnostic tool. Plain radiographs and advanced imaging techniques such as ultrasonography, magnetic resonance imaging (MRI) and CT help localize the anatomic location of the foreign body and vital structures in its proximity [2,4,7,11].

Computed tomography scanning is an excellent modality and is considered the gold standard in foreign body imaging [6]. It has an excellent ability to identify both radiopaque and radiolucent foreign bodies [7]. Furthermore, adding a contrast medium can enhance the 3D visualization of soft tissues and vital structures that lie in close proximity to the foreign body. In the present case, foreign body dislodgement was witnessed clinically and further treatment was planned based on the patient’s medical history and imaging findings. Initial radiographic examination with OPG showed the location of the mini-screw within the soft tissues lying posterior to the right mandibular ramus (Figure 2). This was confirmed by a contrast-enhanced CT, which further helped localize the foreign body to the right lateral pharyngeal space and revealed its proximity to major blood vessels of the neck (Figure 3). A supplementary chest radiograph was obtained to rule out aspiration or swallowing of the mini-screw and turned out to be unremarkable (Figure 1).

The surgical approach to retrieving foreign bodies in the head and neck region is largely dependent upon the anatomic site where it is dislodged. Studies have reported the use of intraoral, transcervical and endoscopic surgical approaches to retrieve foreign bodies displaced into the head and neck soft tissues (Table 1). Rajaran et al. reported removal of an embedded metallic bur from the right mandibular retromolar area, following bone removal for third molar extraction, using an intraoral approach [13]. They further reported the use of CT imaging to localize the foreign body prior to retrieval under GA. Similarly, Bhatta and Shreshtha reported removal of a metal rod dislodged into the oropharynx of an eight-year-old child, following blunt trauma, using and intraoral approach under GA and with the help of plain radiographs taken in perpendicular planes, for localization [14]. In a case reported by Giudice et al., the transcervical surgical approach was used under GA to retrieve a mandibular implant that was iatrogenically displaced into the sub-mandibular space [15]. In yet another case of transcervical surgical approach for foreign body retrieval, Burduk reported removal of a broken toothbrush fragment, measuring about 6 centimeters, from the right lateral pharyngeal space of a one-year-old child [16]. This report also mentioned the use of CT for anatomic localization of the foreign body. Endoscopic approaches for head and neck foreign body were reported for retrieval of a metallic ball in a 12-year-old girl [17], and to remove a fractured wooden skewer in an adult [18]. The foreign bodies were displaced to the parapharyngeal space in both of the above cases.

**Table 1:** List of cases reporting displacement to and subsequent retrieval of foreign body from the lateral pharyngeal space

Author	Nature of foreign body	Surgical retrieval by
Coales et al. (1999) [19]	Wooden skewer	Endoscopic approach
Burduk (2006) [17]	Broken toothbrush	Transcervical approach
Bhatta and Shrestha (2011) [15]	Metallic rod	Intraoral approach
Okumura et al. (2015) [10]	Dental needle	Intraoral approach (Fluoroscopy assisted and using a steel wire as reference)
Pradhan and Gupta (2016) [18]	Round metallic foreign body	Endoscopic approach
Reategui (2020) [13]	Metallic foreign body	Transcervical approach (Fluoroscopy assisted)

The lateral pharyngeal space or parapharyngeal space is a potential fascial space in the head and neck region, containing vital anatomic structures such as the carotid sheath and the ninth, 10th and 11th cranial nerves [8]. Although foreign body displacement to the lateral pharyngeal space is not uncommon, the clinical challenge is in accessing the anatomic site during retrieval and the associated risk of iatrogenic injury to the anatomic structures [16]. Table 2 presents a list of cases involving surgical removal of foreign body from the lateral pharyngeal space, identified based on a review of the literature. While retrieval could be done by either intraoral, transcervical or endoscopic approaches, in the present case, an intraoral approach was used, utilizing the mini-screw entry point as a reference. Additionally, fluoroscopy using a portable C-Arm x-ray imaging system was used with a metallic dissecting forceps as a radiographic reference (Figure 4). Several studies have reported the advantages of using fluoroscopy for intraoperative imaging of foreign bodies, as it enables anatomic visualization in multiple planes [5,9-12]. In most of the above studies, a C-Arm x-ray system was the preferred method of fluoroscopic imaging, as was the case in the present study too. Based on a series of three cases of head and neck foreign body removal, AlBilasi et al. reported that the use of fluoroscopy is a promising modality for foreign body retrieval, which enables accurate real-time localization, shorter intraoperative period, lesser surgical morbidity and hastened postoperative recovery [5]. This was evident from the present report, as the patient had an uneventful postoperative period without damage to any associated anatomic structures.

Based on our review, the only reported case of foreign body retrieval from the parapharyngeal space using a fluoroscopy-guided, intraoral surgical approach was that of the removal of a broken needle by Okumura et al. (2015) [9]. In their case, they had utilized an approach through the tonsillar fossa after intentional tonsillectomy to approach the dislodged dental needle fragment. In contrast, our surgical approach was unique by way of a minimally invasive retromolar incision, distal to the mandibular third molar area and approaching the parapharyngeal space, postero-inferiorly, through the pterygomandibular space. To the best of our knowledge, while there are reports of using a similar approach to retrieve displaced dental foreign bodies exclusively from the pterygomandibular space [19], the use of a similar approach for a parapharyngeal space foreign body is not reported. Nevertheless, fluoroscopy approaches to a deep space such as the lateral pharyngeal space invariably mandated the use of a radiopaque, metallic guide, which was a steel wire in the case of the procedure reported by Okumura et al. [9], and a hemostat in our scenario (Figure 5).

Being presented as only a single case and not as a case series is a limitation of the present report. Nevertheless, this case is indeed a rare occurrence and is being reported along with review of relevant literature to share knowledge and information among all spectrum of dental clinicians. Based on our literature review, we could not find any reported cases of TAD screw displacement, specifically to a deep space such as the lateral pharyngeal space. Interestingly, Giudice et al. (2021) reported in their systematic review that the most frequently reported complications associated with orthodontic mini-screw placement as root perforation with loss of vitality and tooth discoloration, inflammation of surrounding soft tissue and, perforation of nasal floor and maxillary antrum [20]. Although oral surgeons frequently come across cases of foreign body retrieval from perioral soft tissue spaces, the present case report would serve as a precaution for general dentists and orthodontists, who are among those who frequently place TAD screws in the dental clinic. In this regard, it should be noted that the mini-screw was dislodged close to major blood vessels in the lateral pharyngeal space, and could have caused life-threatening hemorrhage without timely intervention.

**Figure 6:**
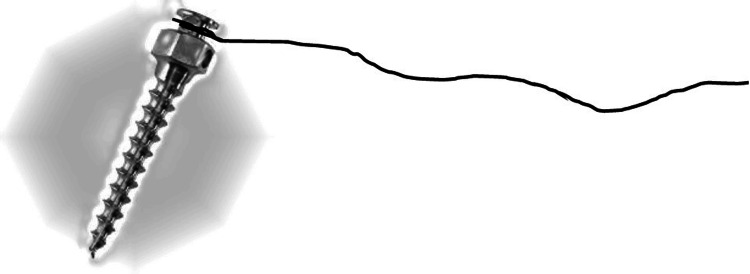
A sterile suture thread tied to the head of the orthodontic mini-screw (TAD) could act as an easy means of retrieval in case of accidental displacement into deeper tissue spaces.

This possible medicolegal complication could be avoided by following proper technique, acquiring adequate training, and adhering to a protocol during TAD screw placement. As a recommendation, the following guidelines may be proposed for avoiding inadvertent displacement of orthodontic mini-screws into deeper soft tissue spaces:

1. Evaluate the anatomical site for mini-screw placement with special impetus on the underlying bone quality and surrounding anatomic spaces and structures [20].

2. Obtain plain radiographs and if needed cone beam computed tomography (CBCT) to ascertain the exact point of mini-screw placement [2].

3. In addition to informed consent, discuss with the patient about the nature of the procedure, its risks and complications, and the need for patient cooperation [3].

4. Administer local anesthesia by infiltration or nerve block and not just topical anesthesia [2,3].

5. Visualize the site for mini-screw placement through optimal mouth opening (aided by a prop), suctioning and illumination [2,3].

6. The mini-screw is inserted through mucosa in a direction perpendicular to underlying bone surface and twisted with gentle pressure until initial few screw threads are embedded in the bone. After which, the screw is inserted deeper in bone by twisting under firm pressure [1].

7. A thin, sterile suture thread (4-0) tied around the head of the mini-screw could aid in the implant retrieval in case of accidental displacement (Figure 6).

8. Exercise extreme caution when attempting placement of mini-screws in areas adjacent to recently extracted sockets and in critical sites such as the mandibular retromolar area, sites close to maxillary tuberosity and infrazygomatic crest, and lingual aspect of the mandible [10,20].

## Conclusions

Orthodontic mini-screws are TADs that are frequently used in the dental clinic and their placement is a routine outpatient procedure performed under local anesthesia. Despite the simplicity of the procedure and its relative ease, care must be exercised during placement as they could inadvertently be displaced as a foreign body into one of the head and neck fascial spaces. Especially, care must be taken while placing mini-screws or implants in the mandibular retromolar area, as they provide a direct path to the lateral pharyngeal space, if and when displaced inadvertently. While it is common for maxillofacial surgeons to deal with foreign bodies dislodged in the head and neck area, proper diagnosis and treatment planning through history, radiographic evaluation and clinical examination is essential before any retrieval attempt. The surgeon must not only be knowledgeable about, but also weigh the risks and benefits of removing the foreign body and the surgical approaches for the same. In this regard, use of intraoperative imaging such as fluoroscopy, promises better surgical outcomes during removal of metallic foreign bodies from the head and neck spaces.
